# Severe Hyperglycemic Crisis: A Presentation of Diabetic Ketoacidosis Requiring Intubation

**DOI:** 10.7759/cureus.106087

**Published:** 2026-03-29

**Authors:** Krina D Patel, Nadiya A Persaud, Michelle Wallen

**Affiliations:** 1 Research, Orlando College of Osteopathic Medicine, Winter Garden, USA; 2 College of Public Health, University of South Florida, Tampa, USA; 3 Emergency Medicine, University of Central Florida Lake Nona Hospital, Orlando, USA

**Keywords:** diabetic ketoacidosis, hyperglycemic crisis, hyperosmolar hyperglycemic state, medication nonadherence, mixed presentation

## Abstract

Diabetic ketoacidosis (DKA) and hyperosmolar hyperglycemic state (HHS) are serious metabolic disturbances that can occur in diabetes, and although they are often described separately, they can share features in clinical practice. This report describes the case of a 62-year-old male patient with type II diabetes mellitus and significant medication nonadherence who presented to the emergency department unresponsive with a Glasgow Coma Scale score of 3. Initial evaluation revealed profound hyperglycemia with a serum glucose level of 1,033 mg/dL, severe metabolic acidosis, an anion gap of 44, and positive ketones, consistent with a mixed DKA/HHS presentation. The patient required emergent intubation, aggressive intravenous fluid resuscitation, insulin therapy, and electrolyte correction. Despite extensive evaluation, no infectious source was identified, and medication nonadherence was determined to be the likely precipitating factor. This case underscores the clinical importance of recognizing overlapping features of DKA and HHS, as reliance on glucose thresholds alone may result in misclassification and delayed treatment. It reinforces the need for comprehensive metabolic assessment in severely hyperglycemic patients and supports viewing DKA and HHS as points along a continuum rather than isolated entities. Additionally, this case highlights the critical role of prevention through diabetes education, medication access, and adherence support to reduce recurrence and improve outcomes.

## Introduction

Diabetic ketoacidosis (DKA) and hyperosmolar hyperglycemic state (HHS) are two acute metabolic emergencies associated with uncontrolled diabetes mellitus and increased counterregulatory hormones such as glucagon, cortisol, catecholamines, and growth hormone. The diagnostic criteria for diabetic ketoacidosis (DKA) include a plasma glucose concentration exceeding 250 mg/dL, the presence of elevated serum ketones, arterial pH below 7.3, and a serum bicarbonate level less than 18 mEq/L [1]. The diagnostic criteria for hyperosmolar hyperglycemic state (HHS) include a plasma glucose level exceeding 600 mg/dL, effective plasma osmolality greater than 320 mOsm/kg, and an absence of significant ketosis [2]. Despite sharing common pathophysiologic mechanisms related to insulin deficiency and hormonal dysregulation, they differ in their biochemical profile and clinical presentation. DKA, classically associated with type I diabetes mellitus, is characterized by hyperglycemia, ketosis, and metabolic acidosis, whereas HHS typically presents with severe hyperglycemia and hyperosmolarity with minimal ketosis in patients with type II diabetes mellitus (T2DM).

Effective management of both DKA and HHS necessitates hospital admission for intensive intravenous fluid resuscitation, insulin administration, and correction of electrolyte imbalances. Administration of intravenous fluids alone helps expand intravascular volume, improve renal perfusion, and lower insulin resistance by reducing circulating counterregulatory hormone concentrations [3]. Equally important is the identification and management of precipitating factors, which most frequently include infections, undiagnosed diabetes, or missed insulin doses [4]. The development of hyperglycemia in DKA can be attributed to a combination of increased gluconeogenesis, enhanced glycogen breakdown, and diminished glucose utilization by peripheral tissues. When insulin levels are insufficient and counterregulatory hormones are elevated, lipolysis is stimulated, leading to the release of free fatty acids into the bloodstream. These fatty acids are subsequently oxidized in the liver to produce ketone bodies such as β-hydroxybutyrate and acetoacetate, ultimately resulting in ketonemia and metabolic acidosis [5]. Events such as stroke or myocardial infarction can induce counterregulatory hormone release while also impairing a patient’s ability to maintain adequate hydration, thereby increasing the risk of profound dehydration, acute kidney injury, and hyperosmolar states [6].

Although traditionally described as distinct entities, emerging literature demonstrates that DKA and HHS may coexist [7]. Studies suggest that mixed DKA-HHS presentations account for greater mortality (8%) than isolated DKA (3%) or HHS (5%), underscoring the clinical severity of overlapping presentations [7]. The diagnostic complexity and increased morbidity associated with these mixed states highlight the importance of early recognition and aggressive management. This case is presented to emphasize the continuum of hyperglycemic crises, illustrate a severe overlapping DKA-HHS presentation precipitated by medication nonadherence, and reinforce the need for heightened clinical suspicion in high-risk patients to reduce preventable complications and mortality.

## Case presentation

**Figure 1:**
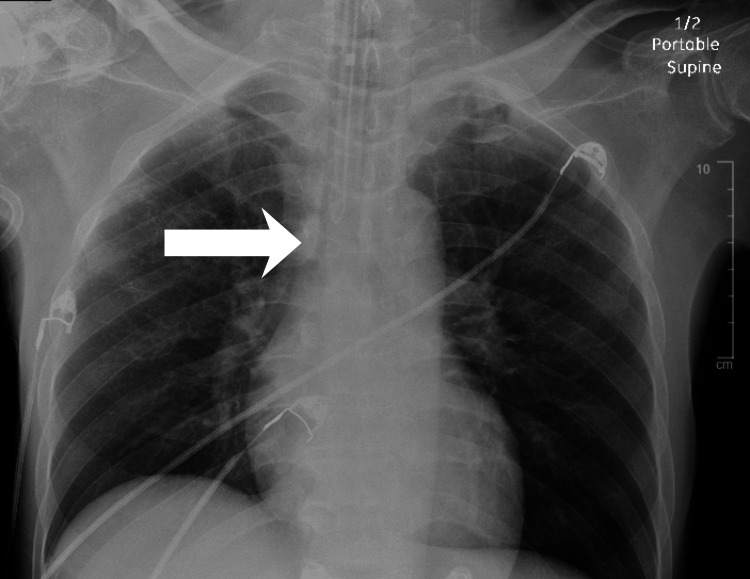
Portable supine chest radiograph on admission demonstrating appropriate endotracheal tube positioning and clear lung fields. The arrow highlights the distal endotracheal tube tip, which is positioned appropriately above the carina. Both lung fields appear clear without evidence of pneumothorax, consolidation, or pulmonary edema, supporting a non-pulmonary cause of the patient’s respiratory failure and altered mental status.

A 62-year-old male patient with a history of coronary artery disease, T2DM, hypertension, left transmetatarsal amputation, and right below-knee amputation was brought to the emergency department unresponsive with a Glasgow Coma Scale (GCS) score of 3 [8]. He was immediately intubated using etomidate and succinylcholine, and correct endotracheal tube placement was confirmed by bilateral breath sounds, end-tidal carbon dioxide (ETCO₂) monitoring, and chest radiography. At the time of intubation, laboratory values, including serum glucose and potassium, were not yet available, and the patient was intubated emergently for airway protection due to a GCS score of 3. Correct endotracheal tube placement was confirmed by bilateral breath sounds, ETCO₂ monitoring, and a portable chest radiograph, which demonstrated appropriate tube positioning and clear lung fields without evidence of pneumothorax, consolidation, or pulmonary edema (Figure 1). These findings supported a non-pulmonary cause of his altered mental status and respiratory failure. Propofol was initiated for sedation. The initial point-of-care glucose measurement was obtained after endotracheal intubation and airway stabilization, at which time it read “high,” raising concern for a hyperglycemic crisis consistent with DKA or HHS.

On presentation, vital signs were: heart rate 81 beats per minute, respiratory rate 22 breaths per minute, blood pressure 107/53 mmHg, and peripheral oxygen saturation (SpO₂) 100%. Despite not meeting full systemic inflammatory response syndrome (SIRS) criteria, a sepsis alert was activated due to the severity of his condition. Intravenous fluids and Rocephin (ceftriaxone) were initiated. The patient’s laboratory results demonstrated severe hyperglycemia and a profound anion gap metabolic acidosis (Table 1). Serum osmolality and beta-hydroxybutyrate levels were not obtained during the initial evaluation.

**Table 1:** Laboratory findings on presentation PCO₂: partial pressure of carbon dioxide; HCO_3_^-^: bicarbonate

Laboratory Test	Patient Value	Reference Range
White blood cell count (WBC)	11.7 ×10³/µL	4.0–11.0 ×10³/µL
Absolute neutrophil count (ANC)	10.07 ×10³/µL	1.5–7.5 ×10³/µL
Hemoglobin	9.3 g/dL	13.5–17.5 g/dL
Platelets	323 ×10³/µL	150–400 ×10³/µL
Blood urea nitrogen (BUN)	82 mg/dL	7–20 mg/dL
Creatinine	4.58 mg/dL	0.7–1.3 mg/dL
Serum glucose	1,033 mg/dL	70–110 mg/dL
Anion gap	44	8–16
Serum CO₂ (total bicarbonate)	3 mmol/L	22–29 mmol/L
Serum acetone	Positive	Negative
Arterial blood gas (ABG)		
pH	6.8	7.35–7.45
PCO₂	14 mmHg	35–45 mmHg
HCO₃⁻	3 mEq/L	22–26 mEq/L

**Figure 2:**
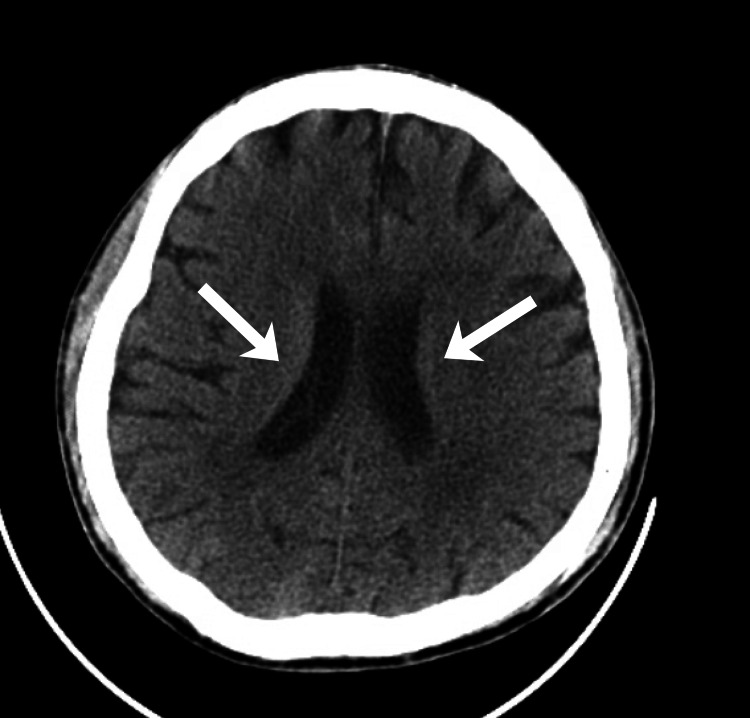
Non-contrast axial CT scan of the head demonstrating normal ventricular size and morphology. The arrows highlight the right and left lateral ventricles, which appear symmetric and without evidence of hemorrhage, mass effect, or cerebral edema. These findings supported the absence of an acute intracranial process contributing to the patient’s altered mental status.

A non-contrast computed tomography (CT) scan of the head was unremarkable, showing no acute intracranial pathology to explain the patient’s altered mental status (Figure 2).

The patient received 2 L of intravenous normal saline, 50 mL of intravenous sodium bicarbonate due to severe acidemia (arterial pH < 6.9), and was started on a continuous insulin infusion prepared as 100 units of insulin in 100 mL of normal saline, titrated per institutional DKA pharmacy protocol. A non-contrast CT of the head was negative for acute intracranial pathology. He was admitted to the intensive care unit for continued management of severe DKA, metabolic acidosis, and acute kidney injury.

The patient’s acute kidney injury (AKI) was managed with aggressive intravenous fluid resuscitation and close monitoring of renal function and urine output. Renal function improved progressively with correction of volume depletion and metabolic abnormalities, and renal replacement therapy was not required.

No evidence of infection was identified. However, the patient’s family later disclosed a history of significant noncompliance with his diabetes medications, which likely contributed to the development of his hyperglycemic crisis.

## Discussion

This case illustrates an increasingly recognized but underreported presentation in which profound hyperglycemia (serum glucose >1,000 mg/dL) coexisted with biochemical and clinical features classically associated with DKA. Traditionally, DKA and HHS are taught as distinct entities, with DKA characterized by marked ketogenesis and metabolic acidosis, and HHS by extreme hyperglycemia and hyperosmolarity with minimal ketosis. However, multiple observational studies and case series have demonstrated that mixed DKA-HHS presentations are not uncommon, occurring in approximately 20-30% of hyperglycemic crises, particularly among older adults with T2DM [9,10]. Similar cases reported in the literature describe patients presenting with severe hyperglycemia exceeding 1,000 mg/dL alongside significant anion gap metabolic acidosis and ketosis, reinforcing the concept that these conditions exist along a clinical spectrum rather than as mutually exclusive syndromes [10]. Our case mirrors these reports, with extreme hyperglycemia, profound acidemia, and ketosis occurring simultaneously in an older patient with T2DM and significant comorbid disease.

The clinical implications of mixed DKA-HHS presentations are substantial. Prior studies have shown that reliance on glucose thresholds alone frequently leads to delayed recognition of DKA in patients presumed to have isolated HHS, contributing to worse outcomes [11]. In this case, despite serum glucose levels exceeding 1,000 mg/dL, the patient exhibited severe metabolic acidosis, a markedly elevated anion gap, and positive ketones, findings consistent with previously described overlap syndromes. Clinicians should therefore assess pH, bicarbonate, anion gap, and ketone status in all patients with hyperglycemia and altered mental status, regardless of absolute glucose values.

Management priorities remain consistent across the spectrum of hyperglycemic crises. Reports of mixed DKA-HHS cases emphasize the need for aggressive yet carefully titrated fluid resuscitation, early insulin therapy, and close electrolyte monitoring, particularly potassium and serum osmolality [12,13]. Fluid resuscitation improves intravascular volume, renal perfusion, and insulin sensitivity by reducing circulating counterregulatory hormones [14,15]. Compared with isolated DKA, mixed presentations often involve larger free-water deficits and higher risks of acute kidney injury, as observed in this patient, necessitating vigilant monitoring to avoid rapid osmotic shifts. Our patient’s acute kidney injury and severe dehydration are consistent with findings reported in prior mixed-crisis cases.

The role of bicarbonate therapy in severe acidemia remains controversial. Randomized and observational studies have not demonstrated consistent benefit from routine bicarbonate administration in DKA, and some suggest potential harm. Guidelines generally reserve bicarbonate for patients with arterial pH < 6.9 or with life-threatening acidemia [16]. Our patient received emergent bicarbonate therapy due to profound acidemia (pH 6.8), a scenario that warrants bicarbonate administration in the setting of extreme acidemia and hemodynamic instability [16,17]. This underscores the importance of adhering to guideline thresholds and carefully weighing potential risks such as hypokalemia, paradoxical central nervous system acidosis, and volume overload [18].

Respiratory compromise is another critical consideration in severe hyperglycemic crises. Case reports of mixed DKA-HHS frequently describe altered mental status, loss of airway protection, and need for early airway intervention, often associated with worse outcomes [19,20]. In this case, a GCS score of 3 necessitated immediate airway control. This aligns with prior reports emphasizing that early, controlled intubation with experienced airway management is essential in profoundly acidemic patients to prevent peri-intubation cardiac arrest [21].

Finally, prevention remains paramount. In our case, family-reported nonadherence was likely a precipitating factor. Recurrent hyperglycemic crises impose substantial clinical and economic burdens, and the literature consistently highlights the importance of structured diabetes education, improved medication access, simplified treatment regimens, and close outpatient follow-up to reduce recurrence and ICU utilization [22]. System-level interventions, including standardized inpatient protocols and comprehensive discharge planning, are essential to mitigating preventable morbidity and mortality associated with hyperglycemic emergencies.

Despite the absence of serum osmolarity and beta-hydroxybutyrate levels, the diagnosis of DKA was supported by the presence of severe hyperglycemia, markedly elevated anion gap metabolic acidosis, low serum bicarbonate, and positive serum ketones. Although the patient’s extreme hyperglycemia (>1,000 mg/dL) raised concern for HHS, the degree of acidemia (pH 6.8) and evidence of ketonemia favored DKA as the primary diagnosis. This presentation is most consistent with DKA, with overlapping hyperosmolar features, a recognized but severe form of hyperglycemic crisis.

## Conclusions

This case emphasizes three important clinical recommendations. First, hyperglycemic crises should be viewed along a continuum rather than as distinct syndromes. Second, elevated serum glucose levels do not exclude a diagnosis of DKA, as demonstrated in this presentation. Third, prevention is critical, and emphasis should be placed on diabetes education, medication accessibility, and adherence support to reduce recurrent hyperglycemic crises and the need for intensive care. Ultimately, early recognition and prompt management are essential to improving outcomes and preventing life-threatening complications. Continued efforts to enhance patient education and close follow-up care are vital in minimizing diabetic emergencies. 
